# Functional brain connectivity prior to the COVID‐19 outbreak predicts mental health trajectories during two years of pandemic

**DOI:** 10.1111/pcn.13654

**Published:** 2024-02-19

**Authors:** María Cabello‐Toscano, Lídia Vaqué‐Alcázar, Ivet Bayes‐Marin, Gabriele Cattaneo, Javier Solana‐Sánchez, Lídia Mulet‐Pons, Nuria Bargalló, Josep M. Tormos, Alvaro Pascual‐Leone, David Bartrés‐Faz

**Affiliations:** ^1^ Department of Medicine, Faculty of Medicine and Health Sciences, Institute of Neurosciences University of Barcelona Barcelona Spain; ^2^ August Pi i Sunyer Institute of Biomedical Research (IDIBAPS) Barcelona Spain; ^3^ Institut Guttmann, Institut Universitari de Neurorehabilitació adscrit a la Universitat Autónoma de Barcelona Badalona Spain; ^4^ Sant Pau Memory Unit, Department of Neurology Institut d'Investigacions Biomèdiques Sant Pau‐Hospital de Sant Pau, Universitat Autònoma de Barcelona Barcelona Spain; ^5^ Department of Medicine School of Medicine and Health Sciences, Universitat Internacional de Catalunya Sant Cugat Spain; ^6^ Departament de Medicina Universitat Autònoma de Barcelona Bellaterra Spain; ^7^ Fundació Institut d'Investigació en Ciències de la Salut Germans Trias i Pujol Badalona Spain; ^8^ Secció de Neuroradiologia, Centre de Diagnòstic per la Imatge, Hospital Clínic de Barcelona Barcelona Spain; ^9^ Centro de Investigación Traslacional San Alberto Magno, Universidad Católica de Valencia San Vicente Mártir Valencia Spain; ^10^ Hinda and Arthur Marcus Institute for Aging Research and Deanna and Sidney Wolk Center for Memory Health, Hebrew SeniorLife Harvard Medical School Boston Massachusetts USA; ^11^ Department of Neurology Harvard Medical School Boston Massachusetts USA

While acknowledging the hardships caused by COVID‐19, the pandemic also provided a unique opportunity to study mental well‐being and individual vulnerability or resilience.^1^, ^2^ Sociodemographic, psychological factors, and lifestyles, have been identified as predictors of mental health during COVID‐19.^3^ Our previous study demonstrated the relevance of the interplay between psychological measures and brain networks' functional connectivity (FC).^4^ However, important questions remain to be addressed. For example, can FC—alone or in combination with other measures – predict longer‐term mental health? Additionally, most studies focus on emotional aspects (psychological distress), although mental health (MH) comprises emotional, psychological (personal growth, [PG]), and social (loneliness) well‐being components, which were differently impacted during the pandemic.^3^ This study aims to investigate if there exists specificity between FC measures and long‐term changes across MH components, knowing the links between brain networks and ‘resilience processes’.^5^, ^6^

We studied 702 healthy, middle‐aged individuals (350 women, age: 50.66 ± 6.98 years) who met the criteria in a 2023 study by Bayes‐Marin and colleagues.^3^ All participants gave written informed consent according to the Declaration of Helsinki. The study protocol was approved by the Comitè Ètic d'Investigació de la Fundació Unió Catalana d'Hospitals (CEIC‐17/06). Resting‐state functional magnetic resonance imaging images acquired before the COVID‐19 outbreak were preprocessed, and system segregation (SyS; integration‐segregation balance) was calculated for seven resting state networks (RSN) [see^4^] and Data **S1**). Multinomial logistic regressions were fitted to predict trajectory membership for the three MH components (Resilient or Chronic trajectories, for psychological distress and loneliness, and Resilient, Progressively Ascending, or Worsening for PG, as captured by growth mixture models contrasting pre‐ versus during‐pandemic observations within two‐year follow‐up (see 3, Data **S1** and Fig. 1a). RSN models included FC for seven RSNs. Full models combined significant RSN measures and significant predictors found in our previous study (age, sex, monthly income, stress coping, personality, general health, and lifestyle habits) (see^3^). Non‐RSN models were as full models but without RSN data, and through likelihood ratio tests (non‐RSN vs full models) we assessed whether the goodness of fit improved by adding FC measures to sociodemographic, psychological, and lifestyle measures. In total, we fitted three models (RSN, non‐RSN, and full) for each of the three MH components.

**Fig. 1:**
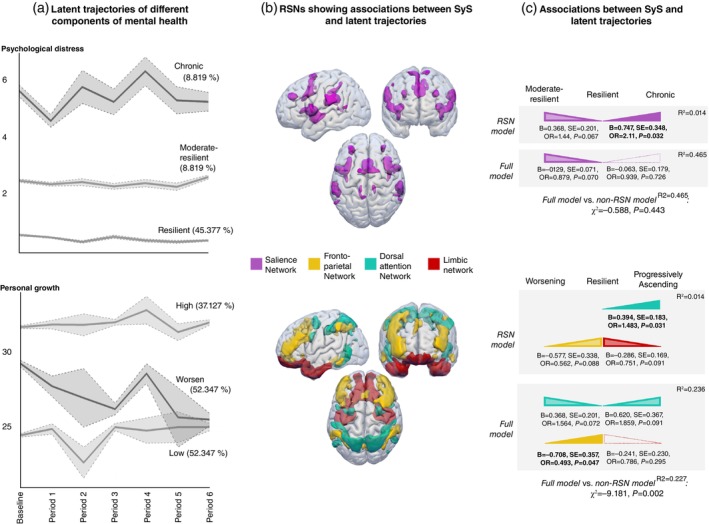
Associations between RSNs SyS and latent trajectories of different components of mental health. Panel (a) depicts the latent trajectories elucidated by Bayes‐Marin *et al*.^3^ subset to the sample of this study. Note that despite three components of mental health being analyzed (i.e., psychological distress, personal growth, and loneliness), only those significant regarding the RSN analyses are displayed. Panel (b) shows a three‐dimensional representation of the brain regions comprising the four particular RSN networks identified in the results section of this study (i.e., Salience, Fronto‐Parietal, Dorsal Attention, and Limbic). Panel (c) describes the associations between SyS values from the networks in B, and the outcomes in A, as estimated by multinomial logistic regressions. Colored triangles indicate the direction of the association between the outcome and RSN SyS values with the same color in B. As Resilient groups were fixated as references in the logistic models, then triangles indicate whether there is a higher or lower probability to belonging to the reference group when SyS increases. Fully colored triangles indicate high significance (i.e., *P* < 0.05), those with thick borders but less opacity indicate marginal effects (i.e., *P* < 0.1), and empty triangles denote effects that were significant in the RSN model that were lost in the full model. Finally, results from the comparison between Full models and non‐RSN models are included. This comparison is performed by likelihood ratio tests, with negative χ^2^ values denoting that the full model is significantly better than the non‐RSN model. OR, odds ratio; PG, personal growth; RSN, resting state network; SE, standard error; SyS, system segregation.

The emotional trajectory membership (Fig. 1a) was significantly predicted by the Salience Network (SN) FC (Fig. 1b), revealing that a more functionally integrated SN (i.e., lower SyS) was more representative of Resilient trajectories in comparison to Chronic and marginally to Moderate‐Resilient ones. In the full model, the significance of SN‐FC as a predictor was reduced, and the comparison of full versus non‐RSN models was non‐significant (Fig. 1c).

The psychological trajectory membership (Fig. 1a) was significantly predicted by the Dorsal Attention Network (DAN), and marginally by Limbic Network (LN) and Fronto‐Parietal Network (FPN). However, in the full model, only FPN‐FC remained strictly significant, with a higher probability of belonging to the resilient trajectory with a greater integrated FPN. Notably, the full model was significantly better than the non‐RSN model (Fig. 1c).

Finally, social trajectory membership was not significantly predicted by any of the RSN‐SyS values (*R* ^2^ = 0.008).

Our findings indicate that measures of FC reflecting the integration‐segregation of principal RSNs offer distinct predictions for long‐term MH outcomes across the COVID‐19 pandemic. This study suggests that pandemic anxious‐depressive trends were affected by SN‐SyS. However, emotional trajectories were predicted by a simpler and equally informative model that did not include RSN information, suggesting that lifestyles and psychological factors are enough to describe them. Nevertheless, when studying PG, baseline FPN‐SyS was shown to add meaningful information to the model derived from aggregated sociodemographic, psychological factors, and lifestyles. Individual differences in PG maintenance likely reflect the capacity to thrive through reappraising and attaching value to stressful situations.^7^ As such, the associations found between PG and FPN connectivity, commonly linked to cognitive flexibility and control processes,^8^ may reveal the importance of cognition within psychological aspects. Finally, not finding any RSN‐SyS associated with social well‐being may be due to measuring the individual's subjective perception but not the direct engagement in social contacts, and/or the previously reported paradoxical effects of the outbreak on loneliness during the outbreak.^9^ Altogether, the present findings contribute to our previous observations,^4^ revealing the impact of the DMN‐ and FPN‐SyS on emotional trajectories through stress‐perception. This supports the triple‐network perspective.^5^, ^6^, ^10^

Overall, our findings suggest that assessing brain network integration versus segregation aids in predicting individual resilience and vulnerability across MH dimensions, allowing for early identification of at‐risk individuals, and the design and evaluation of personalized preventive strategies.

## Disclosure statement

Author APL discloses consulting fees received from Neuroelectrics, TetraNeuron, Medrhythms, and Magstim, where he serves as a paid member of the scientific and medical advisory committees. APL is also listed as an inventor on several issued and pending patents related to the real‐time integration of transcranial magnetic stimulation with electroencephalography and magnetic resonance imaging. These patents encompass applications of noninvasive brain stimulation in various neurological disorders, as well as digital biomarkers of cognition and digital assessments for the early diagnosis of dementia. Additionally, APL acknowledges participation on a Data Safety Monitoring Board or Advisory Board for Linus Health and discloses being a co‐founder with ownership of stock in both Linus Health and TI solutions. Authors GC and JSS disclose being granted or contracted by Fundació la Marató de TV3, Alzheimer Drug Discovery Foundation and Spanish ministry of science and innovation.

## Supporting information


**DATA S1.** Supporting information.
