# Supplementary material for: Functional brain connectivity prior to the COVID‐19 outbreak predicts mental health trajectories during two years of pandemic
Source: Psychiatry Clin Neurosci. 2024 Feb 19;78(4):262–4. doi: 10.1111/pcn.13654 (PMC11804909; doi:10.1111/pcn.13654)
Supplement: Supplementary file 1 — DATA S1. Supporting information. [file PCN-78-262-s001.pdf]

SUPPORTING INFORMATION

Functional brain connectivity prior to the COVID-19 outbreak predicts mental health trajectories during two years of pandemic.

Cabello-Toscano et al.

MENTAL HEALTH OUTCOMES

Emotional component – Psychological distress

To assess psychological distress, we used the Patient Health Questionnaire 4 items (PHQ-4) (1), a screening and accurate measurement of core symptoms or signs of depression ('be bothered by little interest or pleasure in doing things', 'be bothered by feeling down, depressed, or hopeless') and anxiety ('feeling nervous, anxious or on edge', 'be bothered by not being able to stop or control worrying'). Participants were asked to indicate how often they have been bothered by four possible symptoms in the last 2 weeks, rated 0 'not at all', 1 'several days', 2 'more than half the days', or 3 'nearly every day'. A score of six or higher represents the cut-off point for a potential case of depression/anxiety (1). However, in our analyses, we used the continuous form where higher scores mean greater psychological distress.

Social component – Loneliness

In the present study, we used the UCLA 3-Item Loneliness Scale (2), as a proxy measure of social well-being. The UCLA items are related to social integration since they refer to the feeling of being excluded or isolated from others (2). In this brief questionnaire, respondents were asked how often they felt that they: lacked companionship, were left out, and were isolated from others, on a 3-level Likert scale coded from 1 'hardly ever', to 3 'often'. Higher scores indicate greater loneliness.

Psychological component – Personal growth

This domain was constituted by 'personal growth', one of the positive functioning variables extracted from the Ryff Psychological wellbeing scale (SPWB) (3,4). SPWB measure consists of 39 items, constituted by six sub-scales evaluating six aspects of positive functioning. Participants are asked to indicate how accurately each item describes themselves by rating on a 5-level Likert scale ranging from 1 'least like me' to 5 'most like me'. Higher scores indicate better positive functioning. In particular, 'personal growth', is constituted by seven items and refers to one’s openness to new experiences and growth.

MAGNETIC RESONANCE IMAGING

Acquisition parameters

Magnetic resonance imaging (MRI) data were acquired in a 3T Siemens scanner (MAGNETOM Prisma) with 32-channel head coil, at the Unitat d’Imatge per Ressonància Magnètica IDIBAPS (Institut

d'Investigacions Biomèdiques August Pi i Sunyer) at Hospital Clínic de Barcelona, Barcelona. MRI session included accelerated multiband sequences adapted from the Human Connectome Project and provided by the Center of Magnetic Resonance Research at the University of Minnesota. For all participants, a high-resolution T1-weighted structural image was obtained with a magnetization prepared rapid acquisition gradient-echo (MPRAGE) three-dimensional protocol and a total of 208 contiguous axial slices obtained in ascending fashion [repetition time (TR)=2400 ms, echo time (TE)=2.22 ms, inversion time=1000 ms, flip angle=8º, field of view (FOV)=256 mm and 0.8 mm isotropic voxel]. Additionally, a high-resolution 3-dimensional SPACE T2 weighted acquisition was undertaken [TR=3200ms, TE=563ms, flip angle=120º, 0.8 mm isotropic voxel, FOV=256mm]. In the same session, they also underwent resting-state functional MRI (rs-fMRI) multiband (anterior-posterior phase encoding; acceleration factor=8) interleaved acquisitions [T2*weighted EPI scans, TR=800 ms, TE=37 ms, 750 volumes, 72 slices, slice thickness=2 mm, FOV=208 mm].

Preprocessing

The rs-fMRI preprocessing pipeline comprised spatial standardization and nuisance correction by making use of functions from FMRIB Software Library (FSL; version 5.0.11; https://fsl.fmrib.ox.ac.uk/fsl/fslwiki/), FreeSurfer (version 6.0; https://surfer.nmr.mgh.harvard.edu) and Statistical Parametric Mapping (SPM12; https://www.fil.ion.ucl.ac.uk/spm/). To start with, the first 10 scans were removed to ensure magnetization equilibrium. After that, all images were field inhomogeneity corrected (FSL topup tool), all scans realigned to a reference image (FSL MCFLIRT) and then standardized into native T1-weighted space (SPM Coregister). Finally, normalization (SPM Normalize) of all fMRI images to Montreal Neuroscience Institute (MNI152) standard space was performed to ensure among-subjects comparability. As for nuisance correction, different components were defined and manually removed from the rs-fMRI images by the "fsl_regfilt" tool implemented in FSL. These components correspond to (i) motion regressors of rotation, translation, and their derivatives, as estimated during scans' realignment, (ii) a drift estimated by a discrete cosine transform (DCT) as a low-pass frequency filter (<0.01), and (iii) signals from white matter (WM) and cerebrospinal fluid (CSF). In order to extract these, CSF and WM masks were obtained from automatic subcortical segmentation of brain volume, based on the existence of an atlas containing probabilistic information on the location of structures (5). This step was part of the FreeSurfer 'recon-all' processing stream, which was run with default parameters, except for the addition of the T2 flag for the improvement of pial surface reconstruction. That is to say, both T1- and T2-weighted images were used for processing anatomical information.

Quality control

All the MRI images were examined by a senior neuroradiologist (N.B.) to detect any clinically significant pathology (none found). Then, all the acquisitions were visually inspected before analysis (M.C.-T. and L.M.-P.) to ensure that they did not contain MRI artifacts or excessive motion.

As head movement may affect rs-fMRI results (see 6-9), in-scanner head motion was considered. In this study, the frame-wise displacement (FWD) mean was calculated for every subject. FWD was computed as in (6), using the vectors of rotation and translation estimated during scans' realignment as part of the preprocessing pipeline.

After preprocessing, the signal-to-noise ratio (SNR) of the rs-fMRI images was calculated voxel-wise by dividing the mean signal by its standard deviation. SNR was averaged for each of the studied resting state networks (RSNs) by masking the SNR voxel-wise images. Average SNR at the sample level were: Default Mode Network, 59.01; Fronto-parietal Network, 65.71; Dorsal Attention Network, 71.19; Salience Network, 56.31; Limbic Network, 28.56: Visual Network, 57.54; Somatomotor Network, 61.57.

System Segregation

This study has a foundation on two previous studies published by our group and using data from the same cohort. Firstly, our objectives were derived from those studies, where the research article by (10) signalled the importance of this metric to predict psychological resilience vs. vulnerability in the context of COVID-19. To be able to directly compare the results together, we aimed to prioritise this analysis, then being able to interpret the results conjointly.

Both this study and the previous one acknowledge the relationship between resilience and particular resting state networks (RSNs). Also, the effective functioning of a network seems to be supported by maintaining the separation of subnetworks while enabling integration between them. This balance between segregation and integration of the networks has been associated with positive outcomes such as better cognition (11,12) and it has been also discussed to play a critical role in resilience, being a potential marker of protection in the context of Alzheimer's Disease (13).

After preprocessing the MRI data, blood-oxygen-level-dependent signal was extracted and averaged across all voxels falling within each region of interest (ROI). Then, ROI-to-ROI rs-FCs were computed as Pearson correlations and subsequently Fisher-z transformed. Negative values were set to zero and autocorrelations were not considered for the calculation of system segregation (SyS), a versatile graph theory-based measure of functional brain network integrity, as expressed in:

$$SyS = \frac{W_{net} - B_{net}}{W_{net}},$$

W_{net} was computed as the average rs-FC connecting all the nodes within the same network, while B_{net} was computed as the average rs-FC connecting nodes of a network to nodes from the rest of the cortex. SyS captures the balance between within-network (W_{net}) and between-networks (B_{net}) rs-FC.

REFERENCES

1. Kroenke K, Spitzer RL, Williams JBW, Löwe B. An Ultra-Brief Screening Scale for Anxiety and Depression: The PHQ–4. Psychosomatics [Internet]. 2009;50(6):613–21. Available from: https://www.sciencedirect.com/science/article/pii/S0033318209708643

2. Rico-Uribe LA, Caballero Francisco Félix and Olaya B, Tobiasz-Adamczyk B, Koskinen S, Leonardi M, Haro JM, et al. Loneliness, Social Networks, and Health: A Cross-Sectional Study in Three Countries. PLoS One. 2016 Jan;11(1):e0145264.

3. Ryff CD. Psychological well-being in adult life. Curr Dir Psychol Sci. 1995;4(4):99–104.

4. Ryff CD, Keyes CL. The structure of psychological well-being revisited. J Pers Soc Psychol. 1995 Oct;69(4):719–27.

5. Fischl B, Salat DH, Busa E, Albert M, Dieterich M, Haselgrove C, et al. Whole Brain Segmentation: Automated Labeling of Neuroanatomical Structures in the Human Brain. Neuron [Internet]. 2002 Jan 31;33(3):341–55. Available from: https://doi.org/10.1016/S0896-6273(02)00569-X

6. Power JD, Barnes KA, Snyder AZ, Schlaggar BL, Petersen SE. Spurious but systematic correlations in functional connectivity MRI networks arise from subject motion. Neuroimage [Internet]. 2012;59(3):2142–54. Available from: https://www.sciencedirect.com/science/article/pii/S1053811911011815

7. Power JD, Mitra A, Laumann TO, Snyder AZ, Schlaggar BL, Petersen SE. Methods to detect, characterize, and remove motion artifact in resting state fMRI. Neuroimage [Internet]. 2014;84:320–41. Available from: https://www.sciencedirect.com/science/article/pii/S1053811913009117

8. Power JD, Schlaggar BL, Petersen SE. Recent progress and outstanding issues in motion correction in resting state fMRI. Neuroimage [Internet]. 2015;105:536–51. Available from: https://www.sciencedirect.com/science/article/pii/S1053811914008702

9. Van Dijk KRA, Sabuncu MR, Buckner RL. The influence of head motion on intrinsic functional connectivity MRI. Neuroimage [Internet]. 2012;59(1):431–8. Available from: https://www.sciencedirect.com/science/article/pii/S1053811911008214

10. Cabello-Toscano M, Vaqué-Alcázar Lídia and Cattaneo G, Solana-Sánchez J, Bayes-Marin I, Abellaneda-Pérez K, Macià-Bros D, et al. Functional Brain Connectivity Prior to the COVID-19 Outbreak Moderates the Effects of Coping and Perceived Stress on Mental Health Changes: A First Year of COVID-19 Pandemic Follow-up Study. Biol Psychiatry Cogn Neurosci Neuroimaging. 2022 Aug;8(2):200–9.

11. Chan MY, Na J, Agres PF, Savalia NK, Park DC, Wig GS. Socioeconomic status moderates age-related differences in the brain's functional network organization and anatomy across the adult lifespan. Proc Natl Acad Sci U S A. 2018;115(22):E5144–53.

12. Chan MY, Park DC, Savalia NK, Petersen SE, Wig GS. Decreased segregation of brain systems across the healthy adult lifespan. Proc Natl Acad Sci U S A. 2014;111(46):E4997–5006.

13. Ewers M, Luan Y, Frontzkowski L, Neitzel J, Rubinski A, Dichgans M, et al. Segregation of functional networks is associated with cognitive resilience in Alzheimer's disease. Brain. 2021;144(7).
